# Stem Cells in Aggregate Form to Enhance Chondrogenesis in Hydrogels

**DOI:** 10.1371/journal.pone.0141479

**Published:** 2015-12-31

**Authors:** BanuPriya Sridharan, Staphany M. Lin, Alexander T. Hwu, Amy D. Laflin, Michael S. Detamore

**Affiliations:** 1 Bioengineering Graduate Program, University of Kansas, Lawrence, Kansas, United States of America; 2 Department of Chemical and Petroleum Engineering, University of Kansas, Lawrence, Kansas, United States of America; University of California, San Diego, UNITED STATES

## Abstract

There are a variety of exciting hydrogel technologies being explored for cartilage regenerative medicine. Our overall goal is to explore whether using stem cells in an aggregate form may be advantageous in these applications. 3D stem cell aggregates hold great promise as they may recapitulate the *in vivo* skeletal tissue condensation, a property that is not typically observed in 2D culture. We considered two different stem cell sources, human umbilical cord Wharton’s jelly cells (hWJCs, currently being used in clinical trials) and rat bone marrow-derived mesenchymal stem cells (rBMSCs). The objective of the current study was to compare the influence of cell phenotype, aggregate size, and aggregate number on chondrogenic differentiation in a generic hydrogel (agarose) platform. Despite being differing cell sources, both rBMSC and hWJC aggregates were consistent in outperforming cell suspension control groups in biosynthesis and chondrogenesis. Higher cell density impacted biosynthesis favorably, and the number of aggregates positively influenced chondrogenesis. Therefore, we recommend that investigators employing hydrogels consider using cells in an aggregate form for enhanced chondrogenic performance.

## Introduction

The Wharton’s jelly of the human umbilical cord is believed to contain mesenchymal progenitor cells [1]. Wharton’s jelly cells (WJCs), when cultured in chondrogenic medium, have been shown to produce elevated expression of cartilage specific genes such as SOX9, collagen II, and aggrecan [2]. Human WJCs (hWJCs) were first introduced to the 3D musculoskeletal tissue engineering literature in 2007 [3], and have since emerged as a promising alternate source of cells due to desirable properties such as ease of collection, immunocompatibility, superior tropism, and differentiation potential [4–10].

When chondrocytes are plated in monolayer culture, after several passages they lose their native phenotype and express collagen I, which is absent in articular cartilage [11]. The standard method for culturing cells for chondrogenesis has been pellet culture, in which chondrogenic differentiation is facilitated by the direct cell-cell interaction available in the 3D pellet. Our global hypothesis is that multiple aggregates of cells will be able to provide the benefit of cell-cell interaction relative to non-aggregated cells. Others have reported that microencapsulation of aggregates in a hydrogel has resulted in improved chondrogenic differentiation [12, 13]. 3D embryonic stem cell (ESC) aggregates have been shown to initiate chondrogenic differentiation [14]. Condensation of cells by reduction in intercellular spaces is favorable for chondrogenesis [15], and inhibition of cell aggregation delays chondrogenic differentiation [16].

Several studies have explored the use of rat and human stem cell aggregates for musculoskeletal applications. In particular, Goude et al. [17] reported that MSC spheroids composed of 500–1,000 cells maintained a structure analogous to cartilage condensation, and the effect of chondroitin sulfate encapsulation in MSC spheroids also resulted in increased gene expression of collagen II and aggrecan. Lei et al. [18] reported that MSC spheroids had the therapeutic potential to treat repaired cartilage tissue. When grown as spheroids and supplemented with TGF-β1-encapsulated gelatin microspheres [19], human adipose-derived stem cells (hADSC) were shown to differentiate toward the chondrogenic differentiation pathway, thus exploring large sized aggregates for clinical application [20].

To the best of our knowledge, never before have hWJC aggregates been fabricated in a 3D platform, much less been explored for cartilage tissue engineering applications. Moreover, no other study has compared cell suspension and aggregates side by side with two different cell types. Our overall hypothesis for the current study was that the aggregate groups would outperform the cell suspension (CS) groups in chondrogenesis. We further hypothesized that the effect of the aggregate model on chondrogenesis would be dependent on the number of cells per aggregate.

## Materials and Methods

### Cell culture and expansion

hWJC were isolated from Wharton's jelly of five human umbilical cords obtained from the Lawrence Memorial Hospital with informed consent (Institutional Review Board Lawrence Memorial Hospital Protocol# 08–2, University of Kansas Institutional Review Board, Protocol# 15402), with all births at full term and under normal delivery conditions. Written consent was obtained from the patient and the consent method and cord-harvest for approved for this study by the IRB committee. The cord collection and cell harvest was approved by the IRB specifically for this study. We isolated hWJC according to our previous published protocol [21, 22]. Briefly, the cells were cultured in hWJC media, which was composed of 10% MSC qualified fetal bovine serum (FBS) (Invitrogen Life Technologies, Carlsbad, CA) and 1% Penicillin-Streptomycin (Invitrogen Life Technologies, Carlsbad, CA) in low glucose DMEM (Life Technologies, Grand Island, NY). The medium was changed every other day, and hWJCs were maintained at 37°C with 5% CO_2_ in a cell culture grade incubator. At 80% to 90% confluence, hWJCs were trypsinized with 0.05% Trypsin-EDTA (Life Technologies) and expanded in this fashion up to passage 4 (P4). Cells at P4 from all five cords were pooled into one tube.

Rat bone marrow-derived mesenchymal stem cells (rBMSC) were harvested from the femurs of seven young male Sprague–Dawley rats (200–250 g, Charles River) following a University of Kansas approved IACUC protocol for cellular harvest protocol# 175–08. The femur harvest and cell isolation was approved specifically for this study. The cells were isolated according to a protocol previously reported by our lab [3]. Briefly, isolated cells were cultured in rBMSC media (αMEM supplemented with 10% MSC qualified FBS (Life Technologies, Grand Island, NY) 1% Penicillin-Streptomycin (Invitrogen Life Technologies, Carlsbad, CA) and passaged at 80% confluence until P4. All cells from different femurs were pooled together at P4, and used for the study.

The rationale for use of rat BMSCs instead of human BMSCs is for reproducibility, i.e., the opportunity to procure rats of the same strain, age and gender, thus removing the inherent variability associated with adult human BMSC donors. With umbilical cords all coming from patients of the same age (i.e., birth), this concern of age variation that applies to BMSCs does not apply to WJCs. The objective of the study was not to make broad general conclusions about WJCs versus BMSCs, but to rather to explore whether the aggregate method may lead to different results when different sources of mesenchymal cells are used, including their respective different species and culture medium compositions prior to encapsulation in agarose.

### Cellular aggregate formation

**Fig 1:**
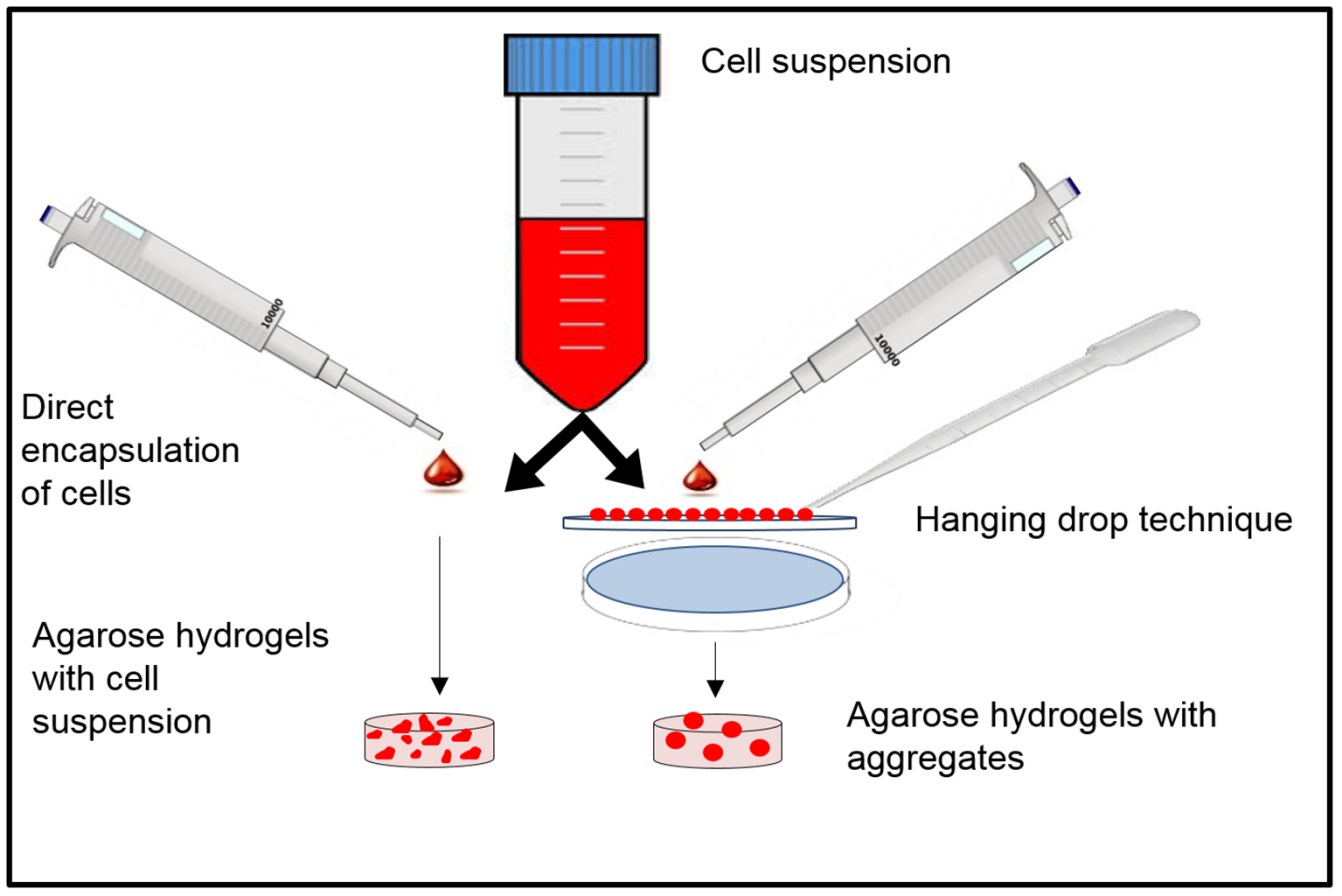
Schematic representation of the hanging down approach and aggregate-encapsulation in agarose hydrogels. The aggregates/CS is encapsulated in pre-polymer solution and undergoes thermal crosslinking to form the hydrogel.

Aggregates for the current study were generated by the hanging drop technique [23]. Cellular suspensions (hWJC & rBMSC) were prepared at two concentrations, either 10.0×10^6^ or 20.0×10^6^ cells/mL, in respective media. Droplets of cell suspensions at a controlled volume of 10 μL that had 10,000 or 20,000 cells, respectively, were pipetted in an array onto the inside surface of a petri dish lid using a 10 μL pipette (Eppendorf, Hauppauge, NY), making sure that the droplets had a safe distance between each other to prevent collision. The cells were allowed to aggregate overnight, aided by gravity when the petri dish was reversed (Fig 1). The petri dish bottom was then filled with PBS to prevent drying of these droplets. After 24 hours, the cell aggregates were collected from the dish with a 1 mL pipette and then encapsulated in agarose hydrogels as described below.

### Encapsulation of cells in agarose hydrogels

Phosphate-buffered saline and low temperature gelling agarose (Type VII- A#A0701) were purchased from Sigma-Aldrich (St. Louis, MO). All other chemical reagents and solvents were of analytical reagent grade. Agarose was prepared according to a previously reported protocol [24]. Briefly, 0.3 grams of agarose was added to 10 mL of PBS and autoclaved for 30 minutes to create a 3% (w/v) agarose solution. The agarose solution was cooled to 39°C after autoclaving. Next, CS or aggregates were pipetted into the agarose solution accordingly. Table 1 gives a comprehensive list of all of the groups, controls, and the respective time points for analysis. For the “low aggregate number” group, one to four aggregates were encapsulated in a single agarose hydrogel, and for the “higher aggregate number” group, five to seven aggregates were encapsulated in a single agarose gel. The aggregates were pipetted into the agarose solution gels very quickly, thus giving only a few seconds of working time and so a few aggregates were always lost in the micropipette tip. To account for the loss in pipetting, a range of numbers (1–4): LA and (5–7): HA was denoted. Using the same number of cells per hydrogel, corresponding controls (cell suspension) were fabricated. For the CS groups, the required amount of cells was pipetted into the agarose solution at 37°C and pipetted gently up and down, then poured into sterile airtight silicone molds (5 mm diameter x 1.9 mm height) and refrigerated at 4°C for 3–4 minutes. The molds were then retrieved, and the constructs were punched out into a 24 well plate (Sigma-Aldrich, St. Louis, MO) and incubated with medium composed of high glucose DMEM (Invitrogen, Carlsbad, CA) with 4.5 g/L d-glucose supplemented with 10% MSC fetal bovine serum (FBS), 1% non-essential amino acids, 1% sodium pyruvate, 50 μg/mL ascorbic acid, 0.25 mg/mL penicillin-streptomycin-fungicide and 100 ng/mL human transforming growth factor (TGF)-β3 (PeproTech Inc., Rocky Hill, NJ) at 37°C and 5% CO_2_. All of the aggregate groups had corresponding CS controls except hWJC 20M LA and HA, which did not have 20M LA CS and 20M HA CS controls, as instead they served the purpose only as comparator groups for the 10M LA and HA experimental groups. Medium was made fresh every other day to avoid freeze-thaw cycles and thus to preserve the bioactivity of TGF-β3, and changed every other day.

**Table 1:** Explanation of the different experimental groups used in the study and their abbreviations.

Study Group	Abbreviation	Control Group	Abbreviation
**1. rBMSC**		**1. rBMSC**	
10 million aggregates (1–4)	10 M LA (lower aggregates/scaffold)	10 million cell suspension (1–4)	10 M LA CS
10 million aggregates (5–7)	10 M HA (higher aggregates/scaffold)	10 million cell suspension (5–7)	10 M HA CS
20 million aggregates (1–4)	20 M LA	20 million cell suspension (1–4)	20 M LA CS
20 million aggregates (5–7)	20 M HA	20 million cell suspension (5–7)	20 M HA CS
**2. hWJC**		**2. hWJC**	
10 million aggregates (1–4)	10 M LA	10 million cell suspension (1–4)	10 M LA CS
10 million aggregates (5–7)	10 M HA	10 million cell suspension (5–7)	10 M HA CS
20 million aggregates (1–4)	20 M LA	Group not included	Group not included
20 million aggregates (5–7)	20 M HA	Group not included	Group not included

### Cell viability assay

To check the viability of the encapsulated cells and aggregates on agarose scaffolds, a fluorescent live/dead viability staining kit (Invitrogen, Carlsbad, CA) was used at days 0 and 21. The samples were washed once with PBS and incubated in live/dead staining solution (0.5 μL calcein and 2.0 μL ethidium homodimer-1 (ETH) diluted in 1 mL DPBS) for 10 min (37°C, 5% CO_2_). The samples were once again washed with PBS prior to imaging using an inverted epifluorescent microscope (Zeiss-LSM 710, Thornwood, NY). Live and dead cells were respectively stained green and red, and the process was repeated on the study groups (aggregates) and the control groups (cell suspensions)

### Biochemical analysis

#### DNA analysis

Constructs were removed from culture in a sterile manner, crushed manually using pipette tips, and incubated in a papain digestion solution consisting of 125 mg/mL papain, 5 mL N-acetyl cysteine, 5 mL ethylenediaminetetraacetic acid, and 100 mL PBS (all reagents from Sigma-Aldrich, St Louis, MO) in distilled water overnight in a 60°C water bath. The next day, sample digests were centrifuged for 5 minutes and stored at -20°C until further use. DNA content was quantified for all samples utilizing a PicoGreen kit (Molecular Probes, Eugene, OR) according to the manufacturer’s instructions. Previous studies [25] from our lab established a conversion factor of 8.5 pg/cell that may be used to convert DNA content to cell number for human cells. For the rat species, the conversion factor of 7.7 pg/cell has been used to convert DNA content to cell number [21, 26].

#### Collagen assay

For quantifying collagen production, a Sircol soluble collagen assay kit and manufacturer’s protocol were used (Biocolor, Belfast, U.K.). Briefly, samples, standards and blanks (distilled water) were incubated in pepsin solution (Sigma-Aldrich) and stored overnight at -4°C. The kit provided standards, and distilled water was used as the blank. 1.0 mL of Sircol dye reagent and 100 μL of each sample were mixed slowly for 30 minutes. Solutions were centrifuged at 12,000 rpm for 10 minutes, and the resulting supernatant was discarded, keeping the pellet intact. The previous step was repeated after the addition of 750 μL of ice-cold acid salt wash. The pellet was resuspended, and 250 μL of alkali reagent was added. Solutions were then vortexed thoroughly and 200 μL of the solution was transferred to a 96-well plate and read at 555 nm in a Multiscan Ascent microplate reader (Thermoelectron Corporation, Waltham, MA). The exact same procedure was repeated for standards and blanks, and all biochemical assays were done in triplicate.

#### Glycosaminoglycan assay

GAG content was measured with the Sircol DMMB assay kit and manufacturer’s protocol (Biocolor B1000 Belfast, U.K.) was followed. Constructs were homogenized with the aforementioned papain digesting solution and left in a 60°C water bath overnight prior to measuring increase in glycosaminoglycan (GAG) content. A chondroitin sulfate standard was provided with the kit, and distilled water was again used as the blank. 1.0 mL of dimethylmethylene blue dye solution was added to 100 μL of sample, standard, and blank. Solutions were mixed slowly for 30 minutes and centrifuged for 10 minutes. The supernatant was discarded, the pellet was resuspended, and 1.0 mL of dissociation solution was added. The solutions were vortexed and transferred to a 96-wellplate and run at 656 nm in a microplate reader.

### Gene expression analysis

Real-time reverse transcriptase polymerase chain reaction (RT-PCR) was used to assess gene expression levels for collagen types I, X; aggrecan; SOX9; GADPH; and Runx2. Collagen II primer was not considered due to high variability in results even after multiple trials. To each sample, 1.0 mL of lysis buffer (Qiagen, Germantown, MD) was added, and after 1 hour, the solutions were homogenized with a QIAshredder column (Qiagen 79656, Germantown, MD) to extract messenger RNA (mRNA) in accordance with the RNEasy Plus Mini Handbook. A high capacity cDNA reverse transcription kit (Applied Biosystems 4368814, Foster City, CA) allowed reverse transcription of mRNA to complementary DNA (cDNA). 10 μL of the master mix and RNA samples were combined in a 96-well plate, which was then loaded into an Eppendorf Realplex Mastercycler (Eppendorf, Hamburg, Germany). cDNA concentrations were normalized with DNASE-free water, and a Taqman gene expression assay kit (Applied Biosystems) provided the seven primers for the above-mentioned genes (Table 2). 1 μL of cDNA from each sample, 10 μL of universal fast master mix (2x), and 1 μL of a specific primer were mixed in a 96-well plate. RT-PCR reactions were then run in an Eppendorf Realplex Mastercycler.

**Table 2:** Summary of the best performing groups for all the functional assays.

Desirable property	Cell type	Best performance group	Time point (weeks)
1. Cell viability			
	rBMSC	20 M LA CS	0
	hWJC	20 M LA CS	0
2. DNA per scaffold			
	rBMSC	20 M HA	2
	hWJC	10 M HA	0
3. GAG per DNA			
	rBMSC	20 M HA	3
	hWJC	10 M HA	2
4. Collagen per DNA			
	rBMSC	20 M HA	3
	hWJC	20 M HA	0
5. Sox-9 and aggrecan gene expression			
	rBMSC	20 M HA, 10 M LA	2 and 3
	hWJC	20 M HA, 20 M LA, 10 M HA CS	3, 3, and 3
6. Collagen II and aggrecan antibody staining			
	rBMSC	10 M LA, 20 M LA, 10 M HA	2, 2, and 3
	hWJC	10 M LA, 20 M HA	0 and 0

### Immunohistochemistry

Frozen hWJC and rBMSC scaffolds were embedded in Optimal Temperature Cutting (OCT) medium (TedPella Inc, Redding, CA) overnight at 37°C and frozen at -20°C until further use. 10 μm sections were generated using a cryostat (MICROM HM 550, Thermo-Fisher, Carlsbad, CA). The sectioned aggregates taken at week 0, week 2, and week 3 time points for both 10M and 20M samples were kept frozen at -20°C prior to staining. Thawed samples were fixed with 5% formalin for 10 minutes, immersed in xylene, rehydrated in graded ethanol, and submerged in 0.1% triton X for 5 minutes (all reagents from Sigma-Aldrich, St. Louis, MO). The sections were exposed to 3% hydrogen peroxide in methanol for 10 minutes to suppress endogenous peroxidase activity, and the slides were immediately incubated in proteinase K (IHCWORLD IW-1101, Woodstock, MD) at 37°C for 10 minutes. Sections were blocked with 3% blocking serum (Vector Laboratories S-2012, Burlingame, CA) for 30 minutes preceding primary antibody incubation for collagen I, collagen II, and aggrecan for 30 minutes (Table 1). Following primary antibody (Lifetechnologies, Carlsbad, CA) incubation where all of the primary antibodies were rabbit IgG, slides were exposed to biotinylated secondary antibody (horse anti-rabbit IgG) and ABC reagent (Vectastain ABC kit PK-6200, Burlingame, CA) for 30 minutes each. Lastly, the ABC reagent was added to the sections after washing with PBS and incubated for 30 minutes and then washed again. Visualization was accomplished with ImmPact DAB peroxidase substrate (Vector laboratories SK-4105, Burlingame, CA) before rinsing with distilled water and counter stained with VECTOR hematoxylin QS stain. Following staining, slides were rinsed in tap water; dehydrated in ethanol, cleared in xylene for mounting (Permount SP15-500 Fair Lawn, NJ), and viewed under an upright microscope (Zeiss, Axiomanager 2.0, Thornswood, NY). Negative controls for IHC consisted of isotype IgG controls (Life Technologies) being added instead of the primary antibody.

### Statistical analyses

All data are expressed as average ± standard deviation. Statistical analyses were performed using one-way ANOVA (Minitab 15, Minitab Incorporated, State College, PA), followed by a Tukey’s post hoc comparison test for repeated measurements. The statistical significance threshold was set at 0.05 for all tests (with p < 0.05)

## Results

### Live-dead assay

#### rBMSC

**Fig 2:**
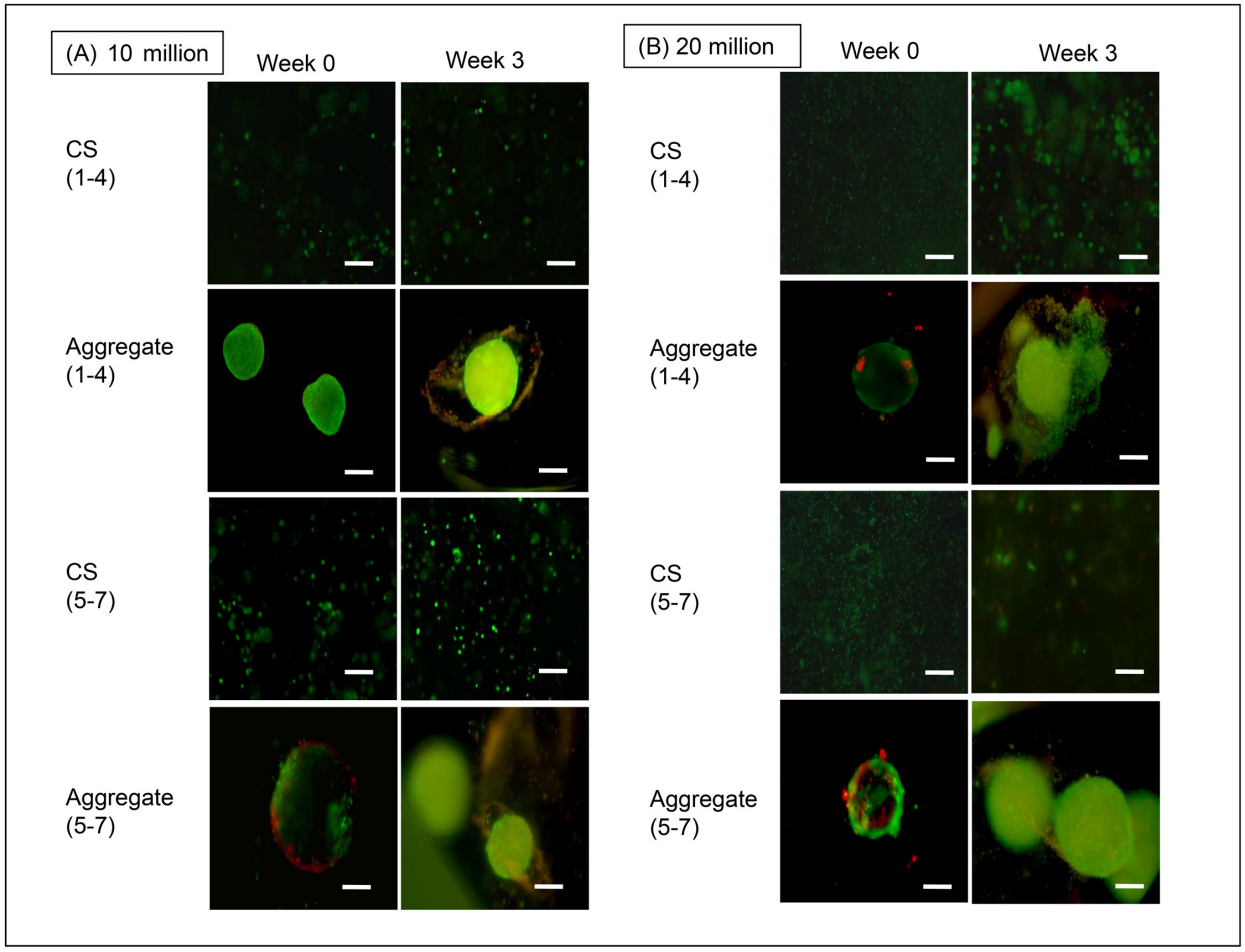
Cell viability at weeks 0 and 3. (A) Live-dead images of the 10M/mL rBMSC aggregates and controls. (B) Live-dead images of the 20M/mL rBMSC aggregates and controls. Scale bar = 100 μm.

Cell viability overall was quite high. Compared to the 20M aggregate groups, the 10M LA and 10M HA grouops had relatively higher densities of live cells at week 3 than at week 0 (Fig 2). 20M aggregates exhibited some cell death right from week 0, and all of the HA groups had more visible dead cells than the LA groups for both 10M and 20M at week 3 compared to week 0. Among the aggregate groups, 20M HA, 10M HA, and 20M LA had condensed regions of cell death at the aggregate periphery at week 0, whereas cell death was distributed throughout the aggregate at week 3. There was no noticeable increase in aggregate diameter over the 3 weeks for any of the groups. There was increased cell death in week 3 in 20M HA CS compared to week 0, but lesser cell death compared to aggregates.

#### hWJC

**Fig 3:**
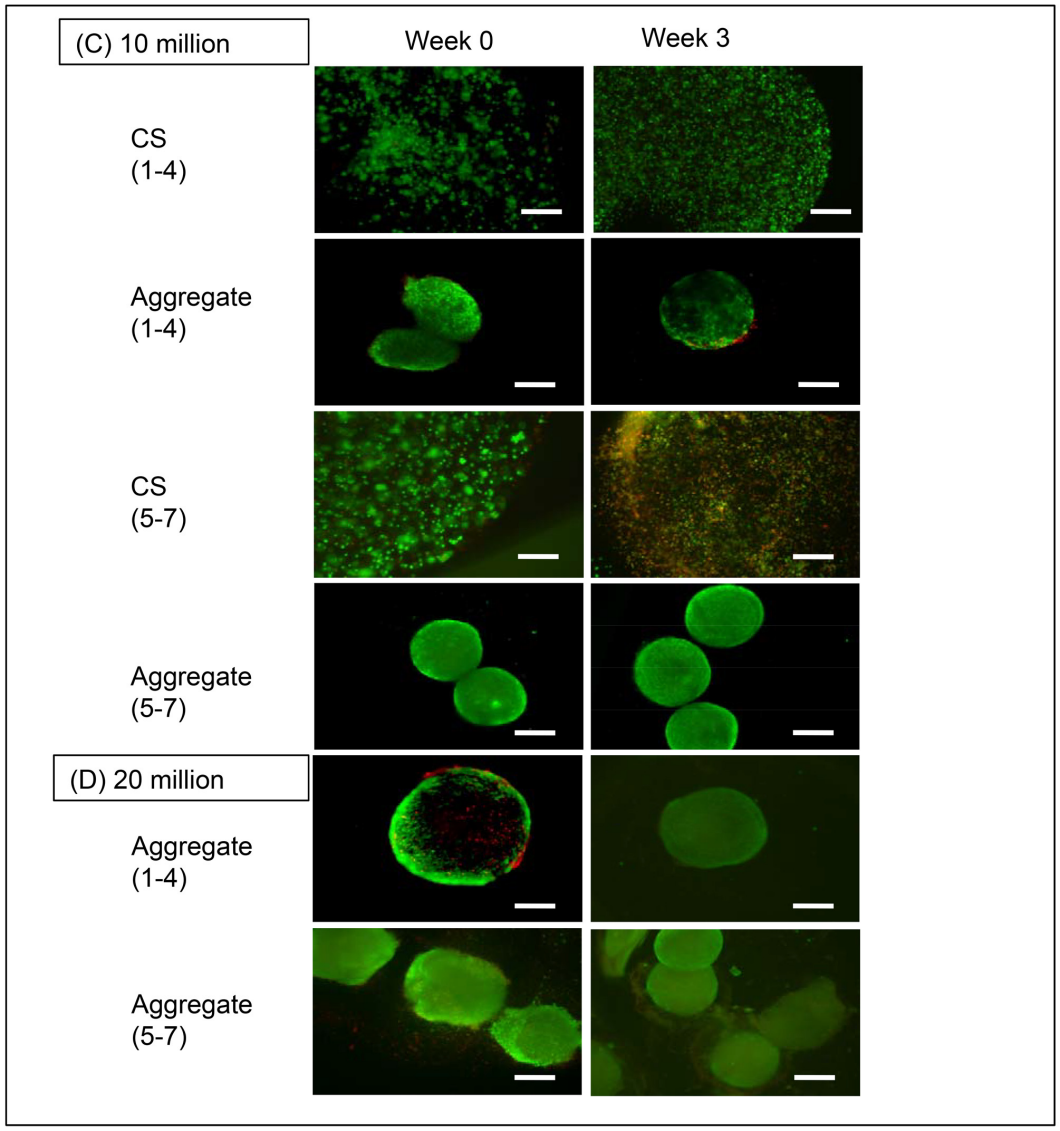
Cell viability at weeks 0 and 3. (A) Live-dead images of the 10M/mL hWJC aggregates and controls. (B) Live dead images of the 20M/mL hWJC aggregates. Scale bar = 100 μm.

The 10M LA CS group showed higher cell viability at week 3 compared to week 0; and 10M HA CS exhibited its highest cell death rate at week 3 (Fig 3). 10M LA and HA maintained consistent cell viability; however, similar to the rBMSC group, the 20M groups experienced relatively more cell death than 10M groups at the week 3 time point. It is also important to note that the 10M HA aggregates displayed a tightly condensed aggregate compared to the LA aggregates. Week 3 images visibly demonstrated an apparent decrease in aggregate diameter and an increase in cell death for 20M LA aggregates. Among the CS groups, the 20M HA and CS groups experienced significant cell death at both week 0 and week 3 compared to the LA CS groups, owing to increased cellular density. Compared to the rBMSC groups, hWJC groups displayed higher viabilities and 20M LA groups were hollow and spherical compared to rBMSC aggregates.

### DNA content

#### rBMSC

**Fig 4:**
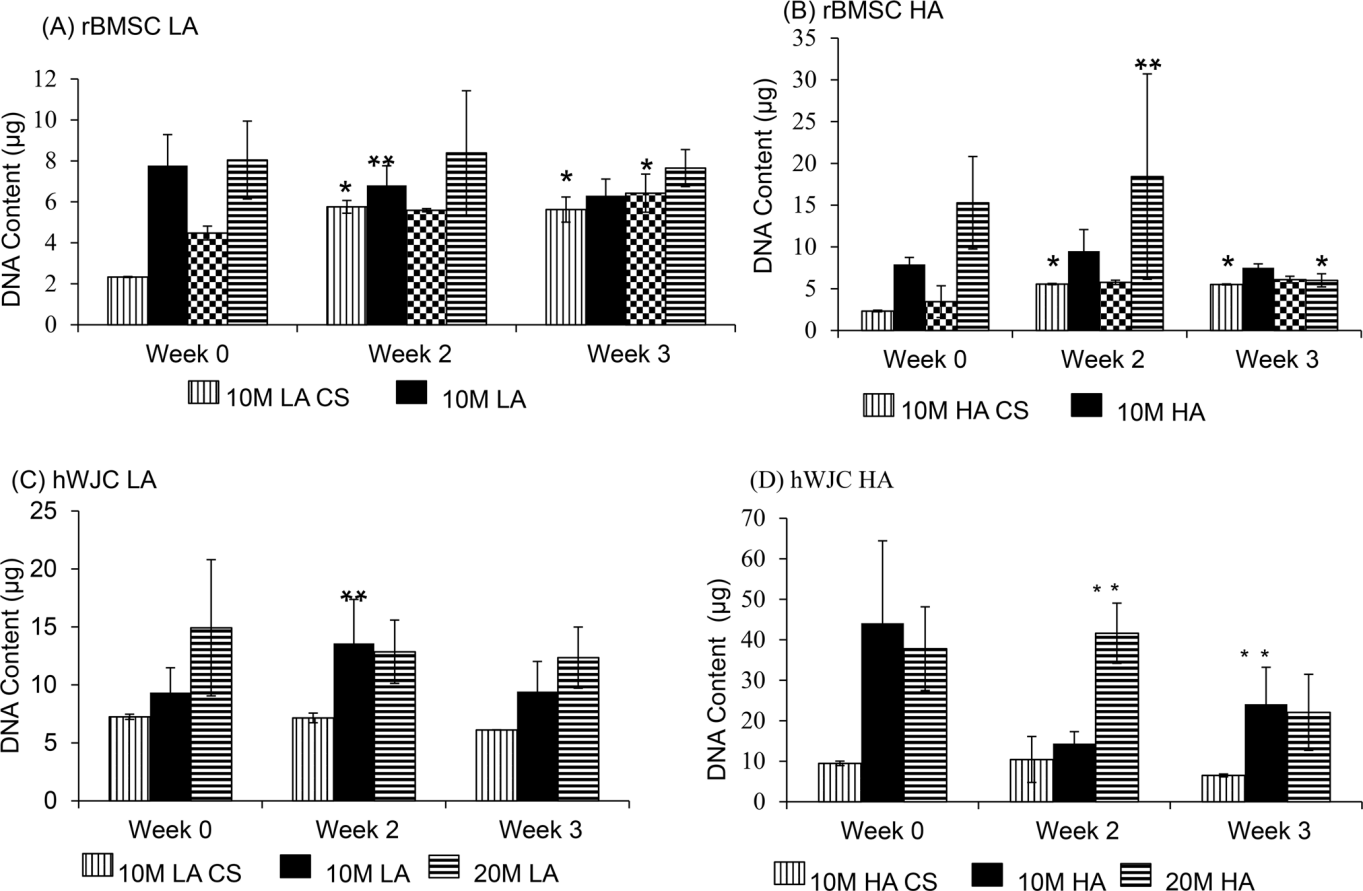
DNA content for all the rBMSC and hWJC groups at 0, 2, and 3 weeks expressed as DNA (μg/ scaffold). (A) DNA content of all the rBMSC LA and LA CS groups. (B) DNA content of all the rBMSC HA and HA CS groups. (C) DNA content of all the hWJC LA and LA CS groups. (D) DNA content of all the hWJC HA and HA CS groups. All aggregate groups had statistically significant increase in DNA over week 0, HA groups had significantly higher values compared to the CS control groups at week 3. Values are reported as mean ± standard deviation, n = 4. (*) represents statistically significant difference from the week 0 value. (#) represents statistically significant difference from the previous time point and (**) represents statistically significant difference from the control at that time point (p < 0.05).

The 10M LA CS and 10M HA CS groups increased in DNA content by 2.6% and 2.3%, respectively, from week 0 to week 3 (p < 0.05). All other CS groups did not display any statistically significant difference in DNA content with respect to time (Fig 4A & 4B). Among the aggregate groups, the DNA content of the 10M LA group decreased by 1.9% from week 0 to week 2 with no significant difference at week 3, and the 20M HA group displayed a 2.5% decrease from week 2 to week 3 (p < 0.05). There were no other significant changes in DNA content over time for the other aggregate groups. In comparing the aggregate and corresponding CS groups, we saw that at week 0, the DNA content of 10M LA aggregates was 2.7-fold greater than the 10M LA CS (p < 0.05), but differences were not significant at other time points.

The differences in DNA contents between the 20M LA and 20M LA CS groups were not statistically significant at any time point.

Among the LA groups, the 10M LA and 20M LA groups did not exhibit any statistically significant differences in DNA content from each other at any time point. On the other hand, the 20M HA group at week 2 had the highest DNA content (18.1 μg) among all of the sample groups (p < 0.05). At week 0, the 20M LA CS group had 1.7% more DNA content than the 10M LA CS group, but there were no significant differences in their values at other time points (p < 0.05).

#### hWJC

Among the CS groups (Fig 4C & 4D), the 10M LA CS, 10M HA CS, 20M LA CS and 20M HA CS groups did not display any significant changes in DNA content over the 3 week period. However among the aggregate groups, only the 10M LA group (Fig 4C) exhibited a statistically significant change over time, with a 1.4% increase in DNA content from week 0 to week 2 (p < 0.05). In comparing the aggregate and corresponding CS groups, the 10M LA group DNA content was 1.6-fold higher at week 2 compared to its 10M LA CS control group, and at week 3 the DNA content of the 10M HA group was 2.6 times higher than that of its 10M HA CS control group (p < 0.05). A comparison between the 10M and 20M groups did not show any significant differences at any time point.

### GAG assay

#### rBMSC

**Fig 5:**
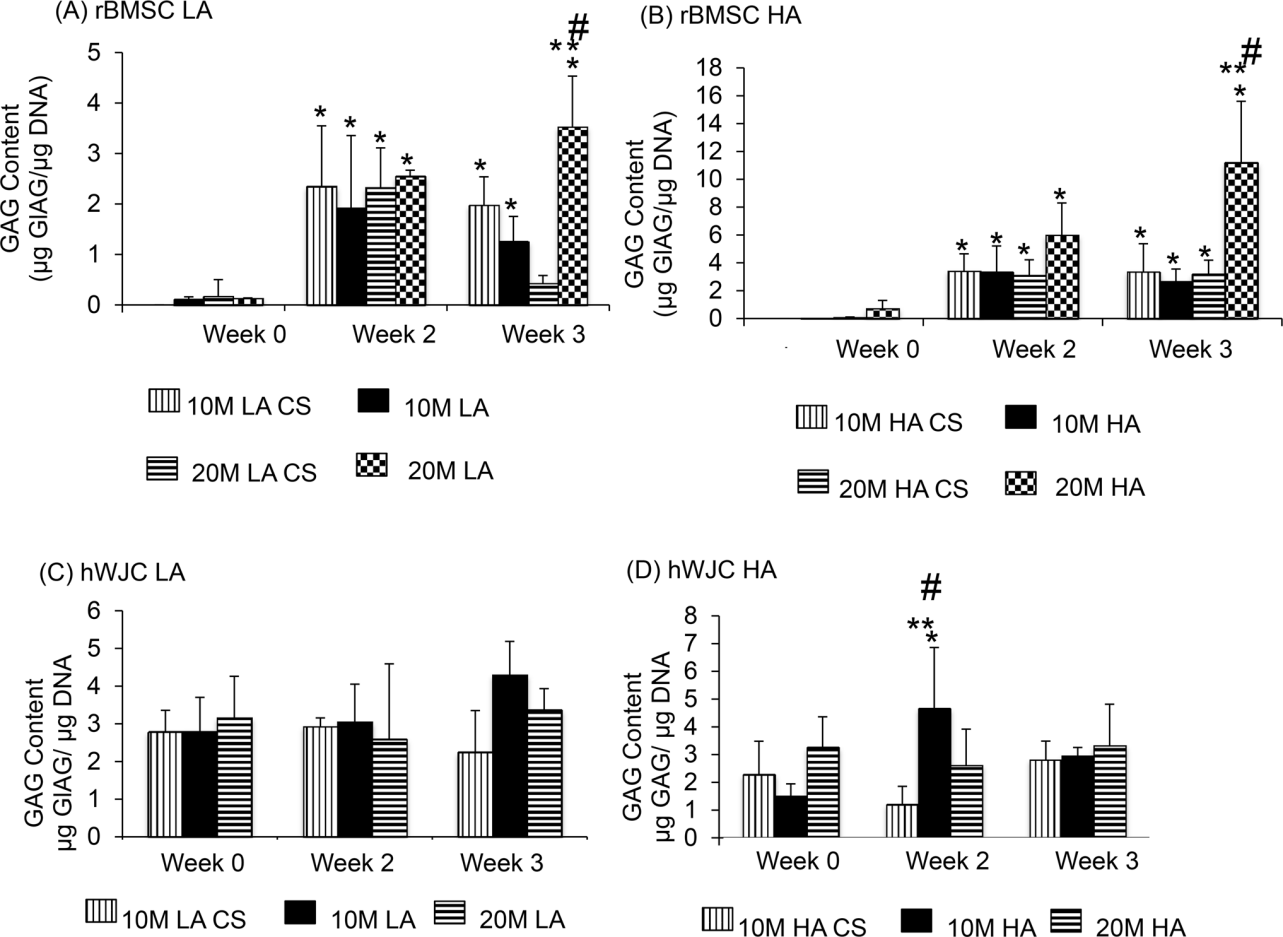
GAG content for all the rBMSC and hWJC groups at 0, 2, and 3 weeks expressed as GAG/DNA (A&B). All rBMSC aggregate groups had statistically significant increase in GAG/DNA over week 0, and select aggregates groups had significantly higher values compared to the control groups at week 3. GAG content for all the hWJC groups at week expressed as GAG/ DNA (C&D) at week 0, 2, and 3. 10 M HA at week 2 exhibited the highest GAG/DNA value and decreased at week 3. Values are reported as mean ± standard deviation, n = 4. (*) represents statistically significant difference from the week 0 value. (#) represents statistically significant difference from the previous time point and (**) represents statistically significant difference from the control at that time point. (p < 0.05).

All values are reported on a normalized (to DNA) basis. Among the CS groups, the 10M LA CS group (Fig 5A) had a 23% increase in GAG/DNA from week 0 to week 2 (p < 0.05) and no significant increase from week 2 to week 3. The 20M LA CS group experienced a 21% increase in DNA content from week 0 to week 2 (p < 0.05), and no other significant differences from week 2 to week 3. The 10M HA CS group had a 43% increase in GAG/DNA content from week 0 to week 2, and similarly the 20M HA CS group had a 60% increase in GAG/DNA content from week 0 to week 2 and no statistically significant increase at week 3 (p < 0.05). Among the aggregate groups, the 10M LA group had a 26% increase in GAG/DNA content from week 0 to week 2 (p < 0.05), but no significant increase from week 2 to week 3. The 20M LA group had a 73% increase at week 2 and 81% increase at week 3 in GAG/DNA content, both relative to week 0 (p < 0.05). The 10M HA group (Fig 5B) had a 43% increase in GAG/DNA content from week 0 to week 2, and the 20M HA group had an 85% increase in GAG/DNA at week 3 compared to week 0 (p < 0.05), although other differences over time for the HA groups were not significant. Comparing the aggregate and corresponding CS control groups, the 20M LA group had a 4.2-fold higher GAG/DNA content compared to its 20M LA CS control group at week 3, and the DNA/GAG content for the 20M HA group was 2.5-fold higher than its 20M HA CS control group at week 3 (p < 0.05). In comparing the 10M vs. 20M groups, 10M HA had 2.7-fold higher GAG/DNA content than 20M HA at week 3 (p < 0.05). Similarly, in comparing the LA vs. corresponding HA groups, the 20M HA group had a 2.3-fold higher GAG/DNA content than the 20M LA group at week 3 (p < 0.05). The 10M LA vs. 10M HA did not display significant differences at any of the time points.

#### hWJC

None of the CS groups (Fig 5C & 5D) showed a statistically significant difference in the GAG/DNA content. Except for the 10M HA group, which had a 21% increase in GAG/DNA at week 2 compared to week 0, no other aggregate group experienced any significant changes over time (p < 0.05). In comparing the control and aggregate groups, only the 10M HA group had a 2.3-fold higher GAG/DNA compared to the control 10M HA CS at week 2 (p < 0.01). All other comparisons, including 10M vs. 20M and LA vs. HA, were statistically insignificant.

### Collagen assay

#### rBMSC

**Fig 6:**
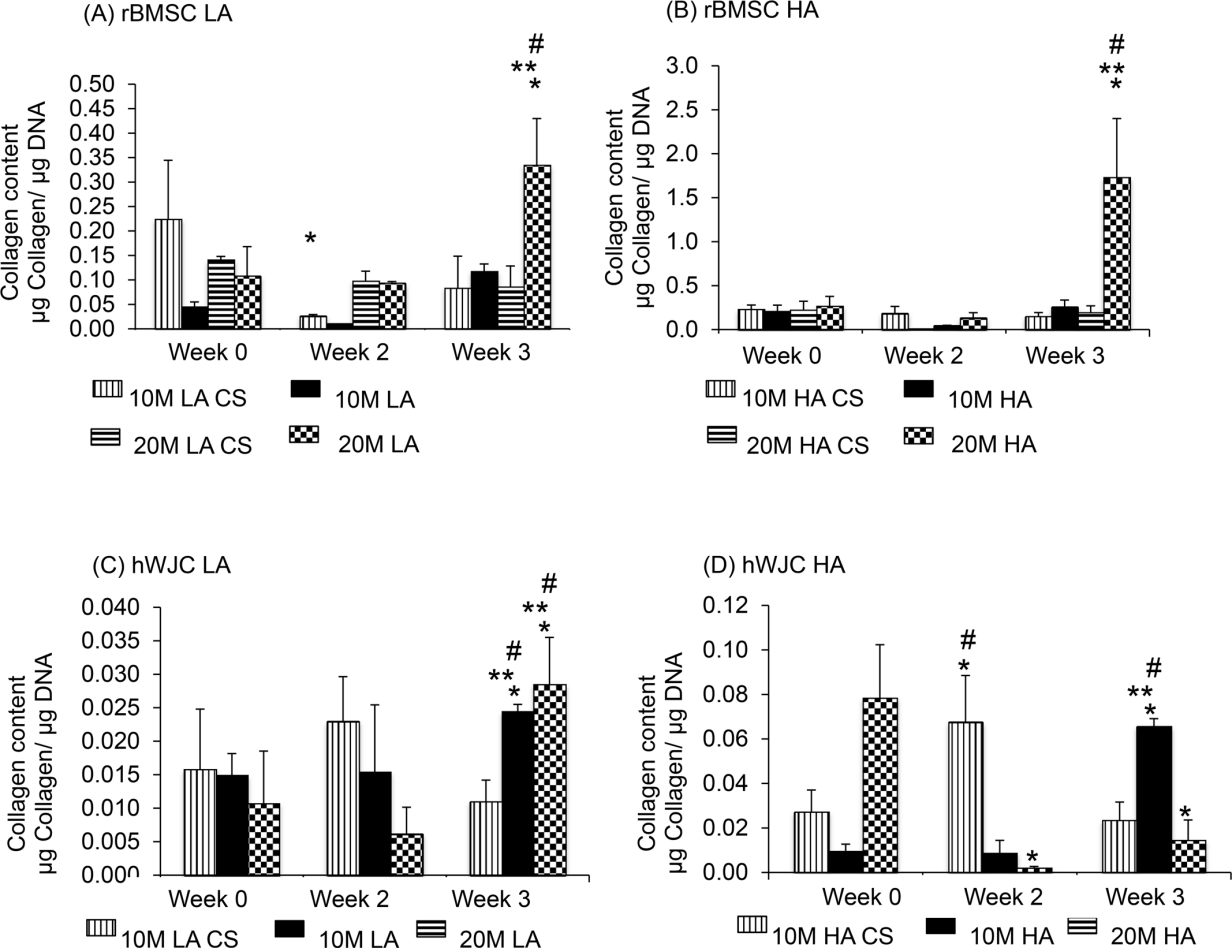
Collagen content for all the rBMSC and hWJC groups expressed as collagen/DNA (A&B) at week 0, 2, and 3. All rBMSC aggregate groups had statistically significant increase in collagen over week 0. At week 3, 20 M HA also displayed the highest collagen/DNA. Collagen content for all the hWJC groups expressed as collagen/DNA (C&D) at week 0, 2, and 3. We noticed that the 20 M HA at week 0 had the highest collagen/DNA value. Values are reported as mean ± standard deviation, n = 4. (*) represents statistically significant difference over the week 0 value. (#) represents statistically significant highest value of the particular group and (**) represents statistically significant difference from the control at that time point. (p < 0.05).

All of the reported values represent collagen content that is normalized to DNA. Among the rBMSC CS groups, only the 10M LA CS group (Fig 6A & 6B) had a significant change in collagen content over time, with a 75% decrease in collagen/DNA from week 0 to week 2 (p < 0.05). Among the aggregate groups, there were two statistically significant changes over time. First, at week 3, the 20M LA group had a 3.8-fold higher collagen/DNA than at week 0, and the 20M HA group had an 8.5-fold higher collagen/DNA content at week 3 than at week 0 (p < 0.05). In comparing the aggregate and corresponding CS control groups, at week 3 only the 20M LA and 20M HA were significantly higher, with 2-fold and 4.6-fold higher collagen/DNA contents relative to their respective CS controls (p < 0.05). In comparing the LA vs. HA groups, the 10M HA group exhibited an 8.7-fold higher and 72% higher collagen/DNA content than the 10M LA group, at weeks 0 and 3, respectively (p < 0.05). Similarly, at week 3, the 20M HA group had a 4.8-fold higher collagen/DNA content compared to the 20M LA group (p < 0.05). Lastly, comparing the 10M vs. 20M groups, we observed that the 20M LA group had a 2.8-fold and 2.3-fold higher collagen/DNA content compared to 10M LA group at weeks 2 and 3, respectively (p < 0.05). At week 3 alone, the 20M HA group had 5.6-fold higher collagen/DNA than the 10M HA (p < 0.05).

#### hWJC

Among the hWJC CS groups (Fig 6C & 6D), only the 10M HA CS group experienced a significant change over time, with a 2.3-fold increase in collagen/DNA content from week 0 to week 2 (p < 0.05). Among the aggregate groups, there were two statistically significant changes over time. First, the 10M LA group at week 3 had a 37% increase in collagen/DNA from week 0 to week 3, and the 10M HA group had a 4.9-fold increase in collagen/DNA content from week 0 to week 3 (p < 0.05). No other groups had a significant increase over time. In comparing the aggregate groups to their respective CS controls, there were four statistically significant differences over the period of 3 weeks. First, the 10M LA group at week 3 alone had a 2.7-fold higher collagen/DNA content than its 10M LA CS group (p < 0.05). Secondly, the 20M LA group at week 3 alone had a 3.1-fold higher collagen/DNA content than its 20M LA CS control group (p < 0.05). In addition, the 10M HA group at week 3 alone had a 2.3-fold higher collagen/DNA content than its 10M HA CS control group (p < 0.05). Finally, the 20M HA group at week 0 had a 63% higher collagen/DNA content compared to its 10M HA CS control group (p < 0.05).

In comparing LA vs. HA, we observed that only at week 3 the 10M HA group had a 2.7-fold higher collagen/DNA content compared to the 10M LA group (p < 0.05). There were no other statistically significant changes over time for any other group. In comparing the 10M vs. 20M groups, there was only one significant difference. At week 0, the 20M HA group had a 7.5-fold higher collagen/DNA content than the 10M HA group, but, at week 3, the 20M HA had a 74% lower collagen/DNA than the 10M HA group (p < 0.05).

## Gene Expression

### rBMSC

#### Collagen I

**Fig 7:**
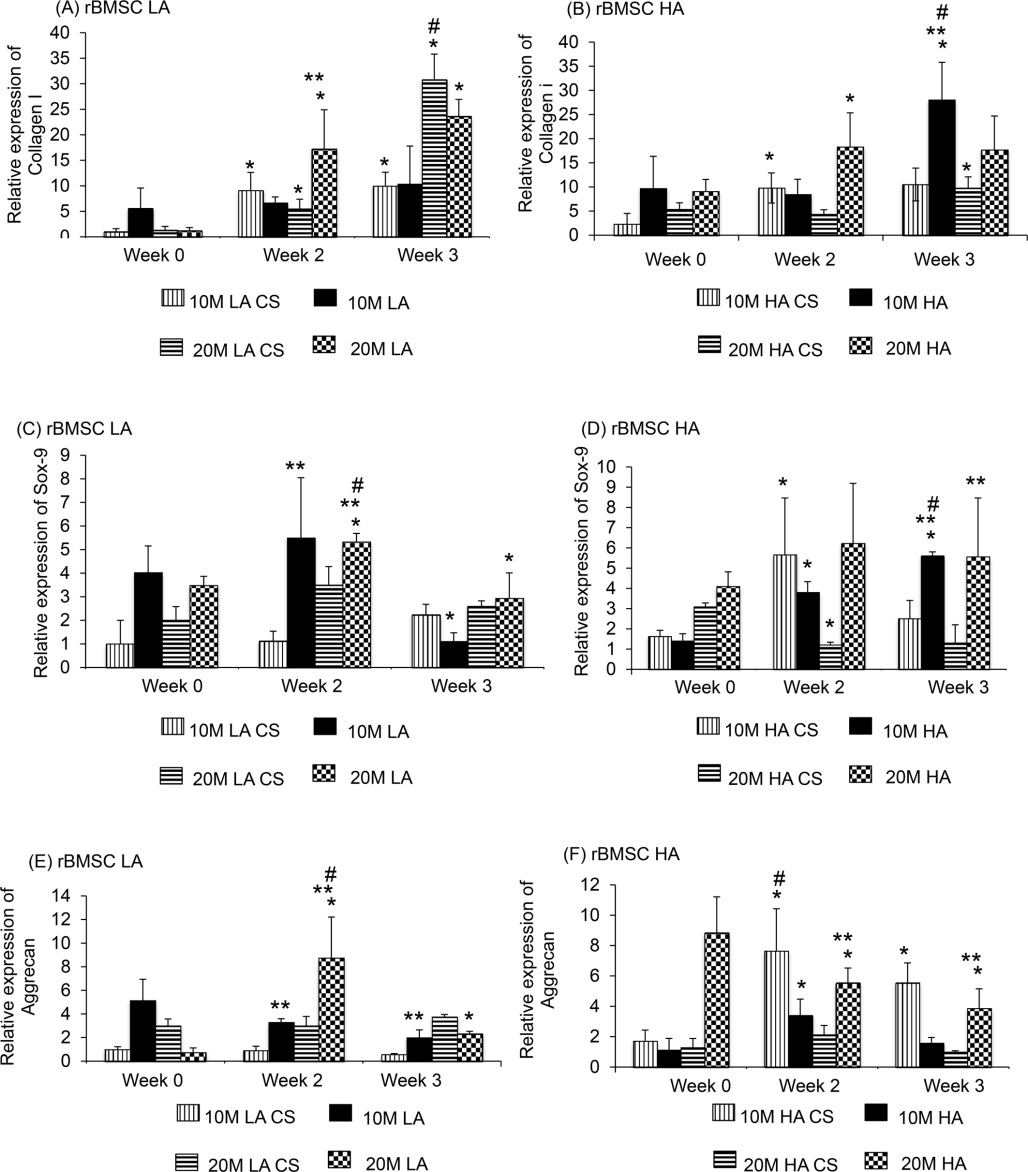
Gene expression of Collagen I, Aggrecan and SOX9 for rBMSC groups at week 0, 2, and 3. There was significant increase in the SOX9 and Aggrecan value at week 2 and 3 for 20 M HA group, compared to the controls. Values are reported as mean ± standard deviation, n = 4. (*) represents statistically significant difference over the week 0 value. (#) represents statistically significant highest value of the group (**) represents statistically significant difference from the control at that time point. (p < 0.05).

The 10M LA CS group (Fig 7A & 7B) had a 3.5-fold increase in expression in collagen I gene expression from week 0 to week 2, and the 20M LA CS group had a 10.2-fold increase in collagen I expression from week 0 to week 3 (p < 0.05). The 10M HA CS group did not show any significant increase in gene expression over time, but the 20M HA CS group had a 97.5% increase in gene expression from week 0 to week 2 (p < 0.05). Among the aggregate groups, there were two significant changes in gene expression over time. First, the 20M LA group had a 5.6-fold increase at week 2 compared to week 0 and no significant increase at week 3 (p < 0.05). Second, the 10M HA group had a 3.6-fold increase in gene expression at week 2 compared to week 0 and no significant increase at week 3 (p < 0.05). The other aggregate groups (10M LA and 20M HA) did not show statistically significant changes over time. In comparing the aggregate and corresponding CS control groups, at week 3, the 10M HA group had a 1.7-fold higher collagen I expression compared to its respective CS control group (p < 0.05).

In comparing the 10M vs. 20M groups, we observed that only at week 0, the 20M LA group had 83% lesser collagen I gene expression than the 10M LA group (p < 0.05). In comparing the LA vs. HA groups, we observed only one significant difference. At week 3, the 10M HA group had a 2.2-fold higher collagen I gene expression than the 10M LA group (p < 0.05). No other differences were significant.

#### SOX9

The 10M HA CS group (Fig 7C & 7D) had a 3.1-fold increase in SOX9 expression from week 0 to week 2, and the 20M HA CS group had a 57% decrease in expression from week 0 to week 2 (p < 0.05). Among the aggregate groups, the 10M LA group had a 4.5-fold increase in SOX9 gene expression from week 2 to week 3, and the 20M LA group at week 2 had a 34% increase in SOX9 expression compared to week 0 (p < 0.05). Additionally, the 10 HA group also had a 27% increase in SOX9 gene expression from week 2 to week 3 (p < 0.05). In comparing aggregate and control groups, we observed that at week 2, the 10M LA group had a 2.8-fold higher SOX9 expression compared to the its CS control group (p < 0.05). At week 2 and week 3, the 20M HA group had a 4.1-fold and 3.8-fold higher SOX9 expression compared to its CS control (p < 0.05), and the 10M HA group had a 1.4-fold higher SOX9 gene expression compared to its CS control group (p < 0.05).

In comparing the 10M vs. 20M groups, we observed only one statistically significant difference. At week 3, the 20M HA group had a 2.6-fold higher SOX9 gene expression than the 10M HA group (p < 0.05). In comparing the LA vs. HA groups, we observed that at week 0, the 10M LA group had a 3.6-fold higher SOX9 expression than the 10M HA group, but at week 3 the 10M HA group had a 5.4-fold higher expression than 10M LA group (p < 0.05).

#### Aggrecan

At week 2, the 10M HA CS and 20M HA CS groups (Fig 7E & 7F) exhibited a 4.0-fold increase and 47% decrease, respectively, in aggrecan gene expression compared to week 0 (p < 0.05), although no changes were significant at week 3. There were no other significant differences in expression by the CS groups at any other time point. Among the aggregate groups, the 20M LA group at week 3 had a 9.0-fold increase in aggrecan expression from week 0 to week 3 (p < 0.01). At week 3, the 20M HA group had a 54% decrease in expression from week 0 (p < 0.05). In comparing the aggregate and respective control groups, the 10M and 20M LA group had a 3.0-fold higher and 2.5-fold higher aggrecan gene expression than their CS control groups (p < 0.05). Similarly, the aggrecan expression in the 20M HA aggregates were 8.3-fold, 2.6-fold, and 3.1-fold higher than the respective CS control group at weeks 0, 2 and 3, respectively (p < 0.05).

In comparing the 10M vs. 20M groups, we observed only one significant difference. At week 0, the 20M HA group had an 8.3-fold higher aggrecan gene expression than the 10M HA group (p < 0.05). In comparing the LA vs. HA groups, we observed that there were two statistically significant differences. First, at week 3, the 10M LA group had a 4.4-fold higher aggrecan gene expression than the 10M HA group (p < 0.05). Lastly, at week 0, the 20M HA group had a 8.6-fold higher aggrecan gene expression than the 10M HA group (p < 0.05). The Collagen X and Runx2 gene expression data were not reported for the rBMSC cell line because the primers led to highly variable results that were not reliable in allowing for comparisons to be made, and thus the data were discarded from the manuscript.

### hWJC

#### Collagen I

**Fig 8:**
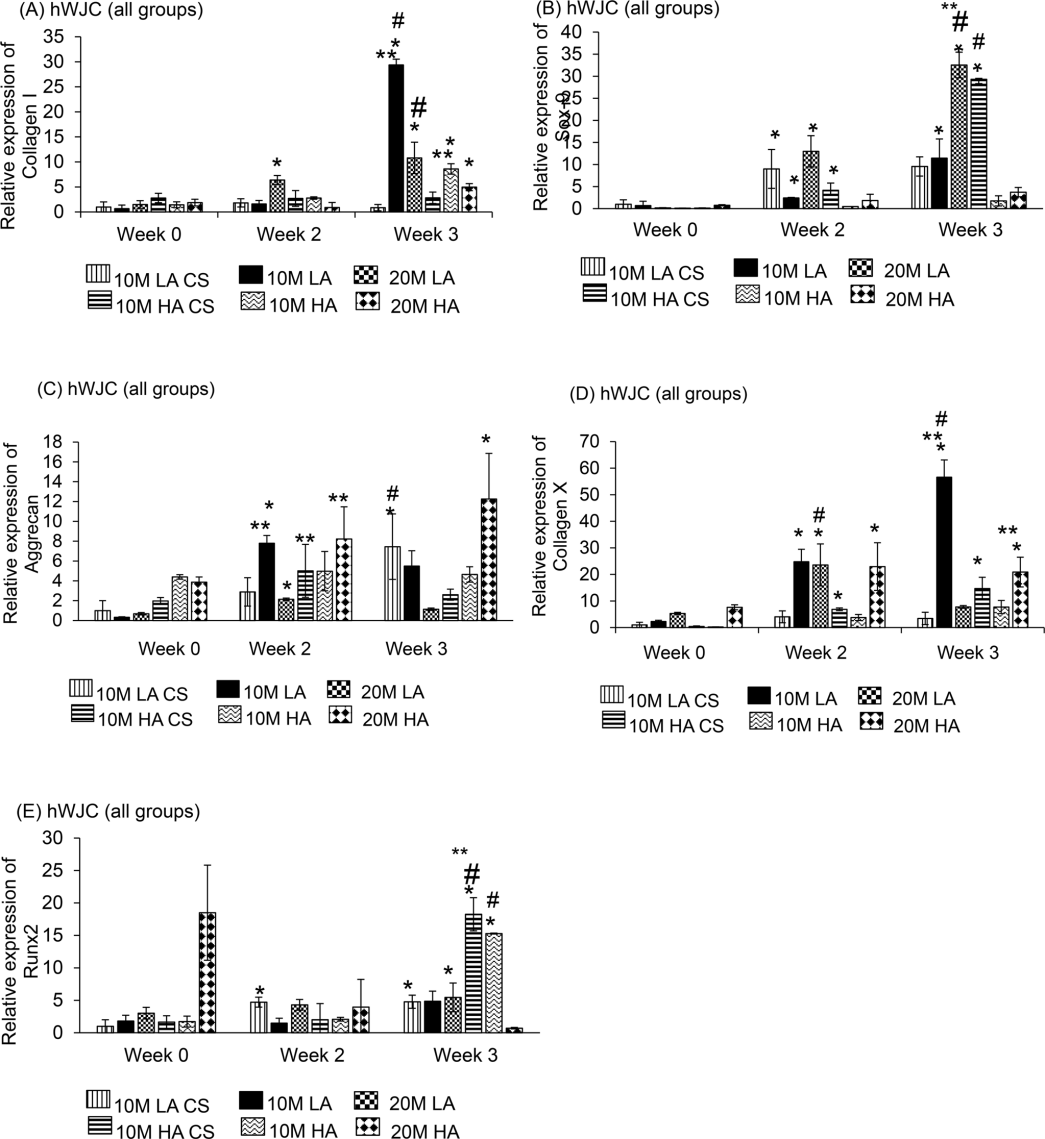
Gene expression of Collagen I, Aggrecan, SOX9, and Runx-2 for hWJC groups at week 0, 2, and 3. hWJC groups at week 0, 2, and 3. There was a significant difference in SOX9 and Aggrecan gene expression at week 3 by groups 20 M HA and 20M HA CS groups. Values are reported as mean ± standard deviation, n = 4. (*) represents statistically significant difference over the week 0 value. (#) represents statistically significant highest value of the group (**) represents statistically significant difference from the control at that time point. (p < 0.05).

The CS groups (Fig 8A) did not have any significant changes in gene expression over the 3-week period. Among the aggregate groups, the 10M HA group had an 8.7-fold increase in collagen I gene expression from week 0 to week 3 (p < 0.05). The 20M LA group had a 4.0-fold and 5.5-fold increase in gene expression at week 2 and week 3, respectively, relative to week 0 (p < 0.05). In addition, the 10M HA group had a 3.5-fold increase in collagen I gene expression from week 0 to week 3 (p < 0.05). In comparing the aggregates to their CS controls, we discovered only one significant difference: at week 3, the 10M LA group had a 10-fold higher collagen I gene expression compared to its CS control (p < 0.05).

In comparing the 10M vs. 20M groups, we observed that at week 2, the 20M LA group had a 2.3-fold higher collagen I expression than the 10M LA group (p < 0.05). However, at week 3, the 10M LA group had a 3.3-fold higher collagen I gene expression than the 20M LA group (p < 0.05). In comparing the LA vs. HA groups, it was seen that only at week 3, the 10M LA group had a 4.8-fold higher collagen I gene expression than 10M HA group (p < 0.05). The comparisons between the 20M LA and 20M HA groups were not significant.

#### SOX9

Among the CS groups (Fig 8B), there was a 4.9-fold increase in SOX9 gene expression from week 0 to week 3 for the 20M LA CS group (p < 0.05). The 10M HA CS group had a 2.2-fold increase in SOX9 gene expression from week 2 to week 3 (p < 0.05). Among the aggregate groups, the 10M LA group had a 5.0-fold higher expression from week 0 to week 3, and the 20M LA group had a 5.6-fold expression from week 2 to week 3 (p < 0.05).

In comparing the 10M vs. 20M groups, the 20M LA group had a 4.2-fold and a 2.3-fold higher SOX9 gene expression than 10M LA group at week 2 and week 3, respectively (p < 0.05). The 10M HA group had a 5.8-fold and a 7.3-fold higher expression in SOX9 compared to 20M HA at week 2 and week 3, respectively (p < 0.05). In comparing the LA vs. HA groups we observed that there were no significant difference in expression between 10M LA and 10M HA at any time point. However, the 20M LA group had a 5.5-fold and a 7.2-fold higher gene expression than the 20M HA group at week 2 and week 3, respectively (p < 0.05).

#### Aggrecan

Among the CS groups (Fig 8C), the 10M LA CS group had a 5.8-fold increase in aggrecan gene expression only from week 0 to week 3 (p < 0.05). The other groups (10M HA CS, 20M LA CS, 20M HA CS) did not show any significant changes over time. Among the aggregate groups, the 10M HA group had a 9.3-fold increase in aggrecan expression from week 2 to week 3, and the 20M LA group had a 6.1-fold increase in aggrecan expression from week 0 to week 3 (p < 0.05). Finally, the 10M HA and 20M HA groups had 4.4-fold and 3.7-fold increases, respectively, in aggrecan gene expression from week 0 to week 3 (p < 0.05).

In comparing the 10M vs. 20M groups, we observed that at week 2, the 10M LA group had 3.1-fold and 2.7-fold higher aggrecan expression than the 20M LA group at weeks 2 and 3, respectively (p < 0.05). In contrast, for the HA groups, the 20M HA group had a 3.3-fold higher aggrecan gene expression than 10M HA group at week 3 (p < 0.05). In comparing the LA vs. HA groups, the 10M HA group had a 4.5-fold higher aggrecan gene expression than the 10M HA group at week 0 (p < 0.05), but no significant differences at weeks 2 or 3. The 20M HA group had 2.7-fold and 13.1-fold higher aggrecan gene expression than the 20M LA group at weeks 0 and 3, respectively (p < 0.05).

#### Collagen X

Among the CS groups (Fig 8D), only the 10M HA CS group displayed a significant change over time, with a 7.5-fold increase in collagen X gene expression from week 0 to week 3 (p < 0.05). The other CS groups (10M HA CS, 20M LA CS, and 20M HA CS) did not display significant changes in expression over time. Coming to the aggregate groups, there was only one significant change in expression. The 10M LA group had 8.3-fold and 2.2-fold increases in collagen X expression from week 0 to week 2, and week 0 to week 3, respectively (p < 0.05). Comparing the aggregate and CS groups, the 10M LA group had 3.5-fold and 10.3-fold higher collagen X gene expression at weeks 2 and 3, respectively, compared to its CS control group (p < 0.05).

In comparing the 10M and 20M groups, at week 3 alone, the 10M LA group had a 5.4-fold higher collagen X gene expression compared to the 20M LA group (p < 0.05). In addition, the 20M HA group had 8.6-fold and a 2.9-fold higher collagen X gene expression compared to the 10M HA group at weeks 0 and 3, respectively (p < 0.05). In comparing the LA vs. HA groups, we observed that the 10M LA group had a 5.8-fold and a 7.8-fold higher collagen X expression than the 10M HA group at weeks 2 and 3, respectively (p < 0.05). The 20M HA group had a 4.7-fold and a 6.3-fold higher collagen X gene expression than the 20M LA group at weeks 0 and 2, respectively (p < 0.05).

#### Runx2

The 10M HA CS group (Fig 8E) had a 2.5-fold increase in Runx2 expression from week 0 to week 2, but no statistically significant increase at week 3 (p < 0.05). The 10M HA CS group had a 6.0-fold increase in Runx2 gene expression only from week 0 to week 3 (p < 0.05). Among the aggregate groups, the 20M HA group had a 97% decrease in Runx2 expression from week 0 to week 3 and the 10M HA group had a 5.6-fold increase from week 0 to week 3 (p < 0.05).

In comparing the 10M vs. 20M groups, we observed that at week 3 alone, the 10M LA group had a 8.3-fold higher Runx2 expression than 20M HA group (p < 0.05). In comparing the LA vs. HA groups, we observed that at week 3 alone, the 10M HA group in turn had a 3.5-fold higher expression than 10M LA group (p < 0.05).

### Immunohistochemistry

#### rBMSC

**Fig 9:**
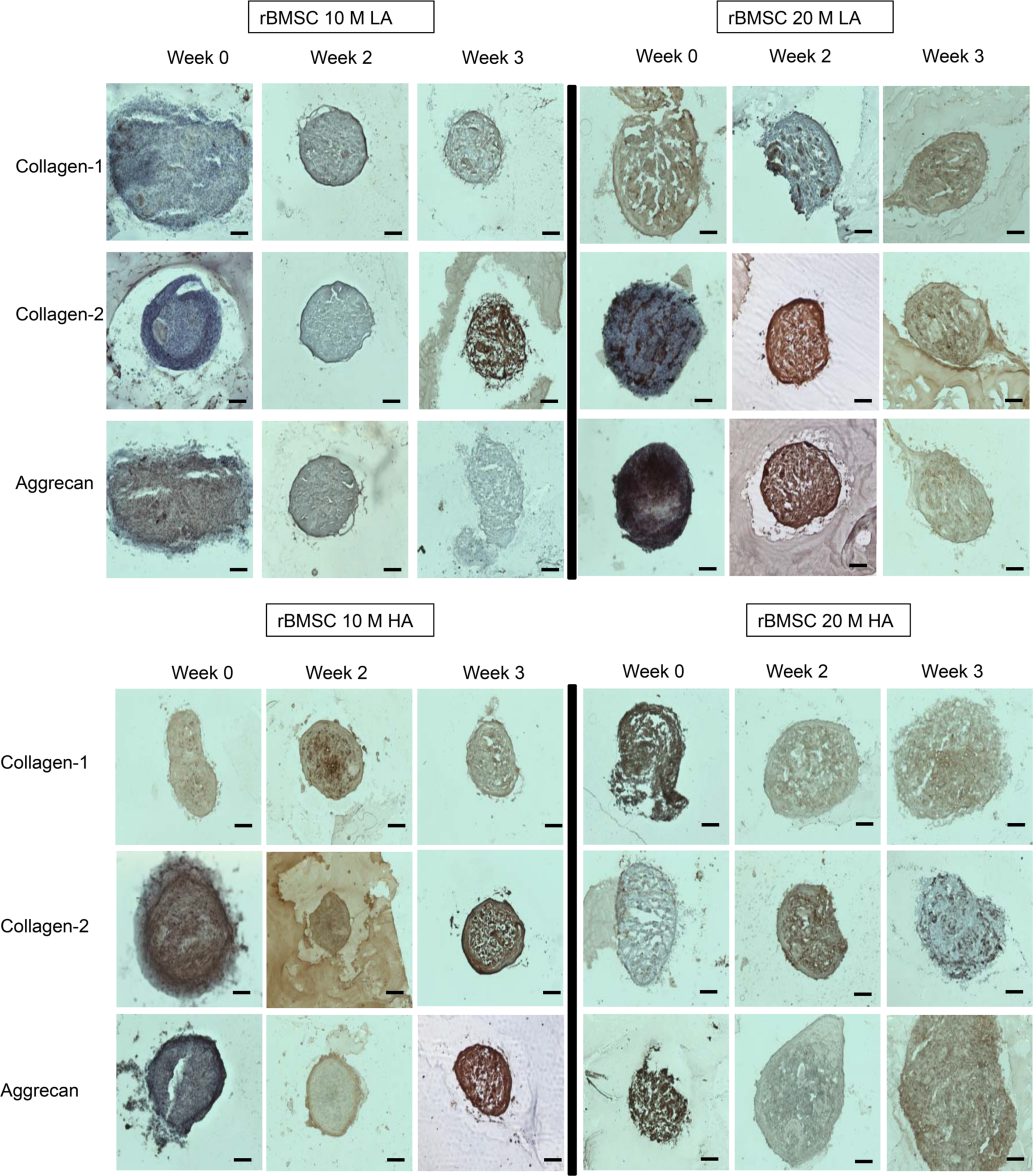
Representative images for immunohistochemistry analysis for collagen I, collagen II, and aggrecan staining for rBMSC groups at weeks 0, 2, and 3. At week 2, 20M LA had the most intense staining for collagen II and aggrecan. At week 3, 10M LA had the highest staining intensity at collagen II. Scale bar = 200 μm.

IHC staining of the rBMSC aggregates revealed that the diameter of the aggregates decreased over the 3-week period, specifically the 10M HA (Fig 9) and 20M LA aggregates. In contrast, the 20M HA aggregates increased in size from week 0 to week 3. Except for the 10M HA group that showed increased collagen I staining at week 2 compared to week 0, collagen I staining did not increase with time for any other group. Collagen II and aggrecan staining were more intense in the 20M group for LA (especially at 2 weeks), but more intense in the 10M group for HA (especially at 3 weeks).

#### hWJC

**Fig 10:**
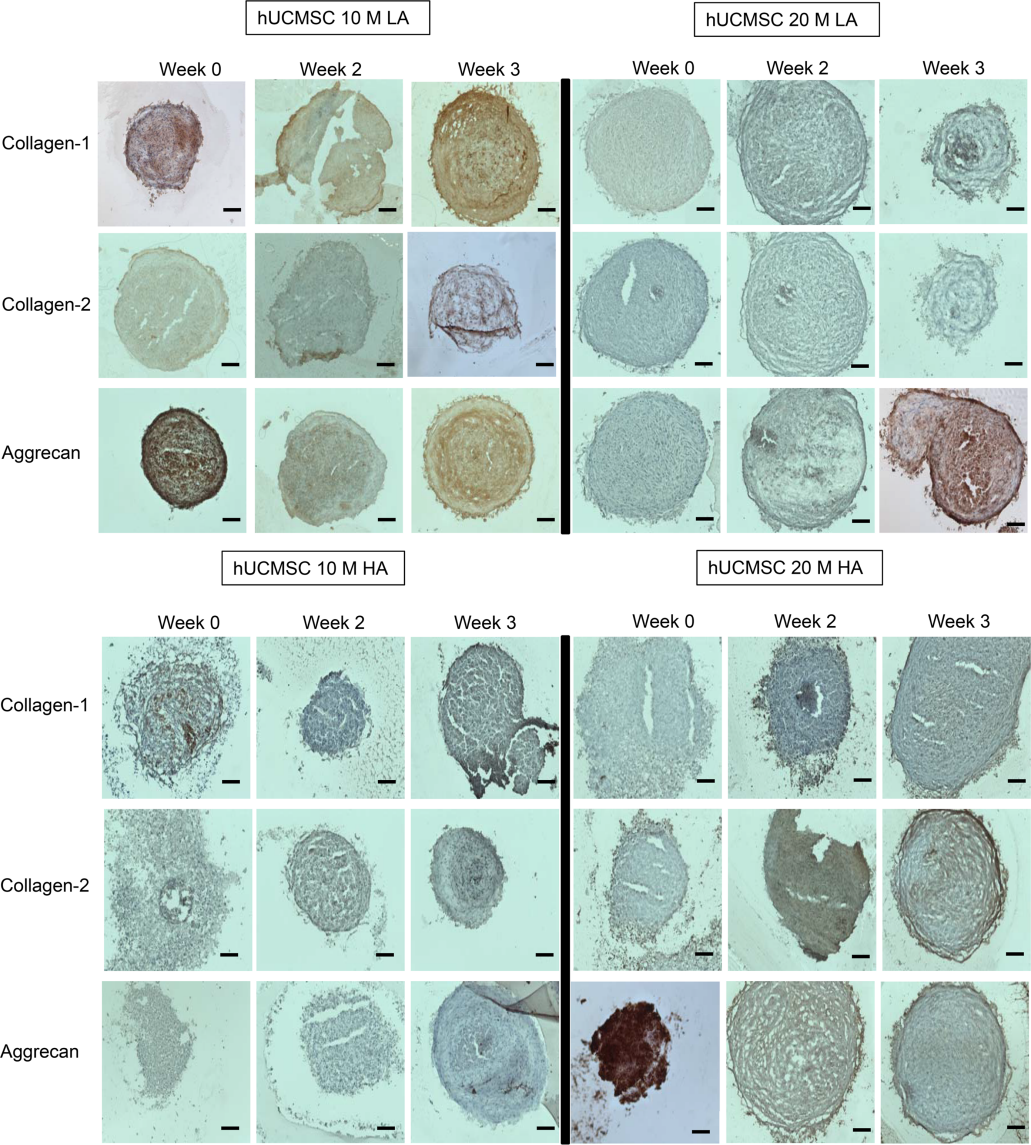
Representative images for immunohistochemistry analysis for collagen I, collagen II, and aggrecan staining for hWJC groups at week 0, 2, and 3. The 10M LA and 20M HA groups at week 0 displayed the highest staining intensity for aggrecan and 20M LA group at week 3 displayed the highest staining intensity for aggrecan. Scale bar = 200 μm.

Collagen I stain was prominent in the center of the aggregate for the 20M LA group at week 3 (Fig 10). Collagen I did not increase in staining intensity over the 3 week period except for the 10M LA and 20M LA group, where the collagen I staining was highest at week 3. 20M HA showed highest collagen II staining at week 3. Aggrecan presented a very interesting staining pattern in the 10M LA and 20M HA groups, as both of these groups had starting aggrecan staining at week 0 to be the most intense (throughout the aggregate) and at week 3, staining was localized to specific patches near the center (10M LA) and around the periphery (20M HA). The 20M LA group were also striking for its intense aggrecan staining at week 3.

## Discussion

This is the first attempt to explore the potential of hWJC aggregates, and also the first to evaluate hWJC and rBMSC aggregates with CS in the context of chondrogenesis. Cell-based approaches have been recently explored to regenerate several organ types, serve as a model for drug testing and routinely used in high throughput screening applications [27–30]. The use of stem cell aggregates for differentiation into the cartilage phenotype is a promising avenue for cartilage regeneration because of the ability to mimic mesenchymal condensation and provision of favorable microenvironment for cell differentiation. Hydrogel-based strategies are especially well poised to capitalize on these advantages by moving away from suspensions of individual cells to aggregate based strategies.

Successful cell-based approaches have been reported using methods such as cell sheet technology, employing human embryonic stem cell aggregates and bioprinting cell-laden microcarriers, and have reported different results with respect to matrix biosynthesis and chondrogenesis [19, 31–33]. Cell-sheet technology has broadly been used in the sense of cell-based approaches, although attempts are now being made to classify them into more refined ways [34]. A few other groups looked into rat and mammalian stem cell aggregates, but did not employ the bone marrow or Wharton’s jelly cell type and the specific aggregate sizes used in the current study was not considered [23, 35, 36] as well. Our group has published several reports on hWJC cells for multiple applications, including chondrogenesis [5, 37, 38], and we are pleased to report that by far, the current aggregate system has produced the highest aggrecan and SOX9 gene expression and corresponding staining for aggrecan IHC compared to our previous studies.

The current study further attempted to understand the influence of the number of aggregates per hydrogel, the number of cells per aggregate, and compared and contrasted the results with the conventional CS approach (Table 2). Lastly, the study served as a great opportunity to compare the chondrogenic performance of hWJC compared to rBMSC, a well-established cell source for musculoskeletal tissue engineering applications [8, 39]. Although the current study may not be able to conclusively prove the mechanisms by which the aggregates consistently outperformed the CS groups, it might be possible that the presentation of cells in a condensed manner allows better intercellular communication and such a high cell to cell interaction may be the basis for chondrogenesis.

The cell viability and DNA content of all rBMSC aggregates had a higher starting DNA content (Figs 3 and 4) at week 0, however, at week 3, the CS and aggregate groups had comparable DNA content, perhaps due to a lack of adequate nutrient supply and space to proliferate. All of the hWJC aggregates followed a similar pattern with the aggregates having a higher starting DNA content compared to the CS controls at week 0, and at week 3 both groups (CS and aggregates) and had comparable DNA content (Fig 4). For the rBMSC group, both LA and HA groups had the same starting GAG/DNA content at week 0. But at week 3, the 20M HA group had a 3 fold increase compared to 20M LA suggesting that the aggregate number had an impact on GAG/DNA biosynthesis. However, the hWJC aggregates did not have any statistically significant increase in GAG biosynthesis compared to the control groups at any time point. On a per DNA basis for the rBMSC group, the 20M LA and 20M HA groups had the highest collagen content (Fig 6) at week 3 compared to their control groups. Taken together, findings reveal that on a per cell basis, both rBMSC and hWJCs did better in matrix biosynthesis and chondrogenesis when in aggregates than in free cell suspension. Secondly, the cell density seemed to impact the biosynthesis (i.e., 20M better than 10M) than for chondrogenesis (i.e., both 20M and 10M groups did well). Moreover, the number of aggregates also mattered more for biosynthesis (i.e., HA outperformed LA) than for chondrogenesis. In the future, it will be necessary to explore which parameter values and aggregate formation methods are most supportive of chondrogenesis, but at least here we have demonstrated that the concept of aggregates to enhance chondrogenesis in hydrogels, with two different stem cell sources, is worth exploring, and that manipulation of parameters can impact the level of improvement.

Some of the limitations in the current study extended to spatial and temporal control of aggregate placement in the agarose gels that may be addressed by a fully automated system that can direct aggregate placement. In the current study, agarose was used merely as a proof of concept method to demonstrate the impact of aggregation on cellular differentiation. In this sense, future studies could use cellular aggregates in conjunction with bioactive hydrogel materials such as functionalized poly(ethylene glycol), hyaluronic acid and other ECM-derived hydrogels to name a few [40–42]. For the current study, it should also be noted that aggregates were directly harvested from the petri-dish and encapsulated into the agarose gels instead of chondrogenically inducing the aggregates. Recently, it has been reported that exposure of aggregates to chondroinductive media prior to encapsulation provides an extremely favorable environment for the aggregates to be “primed,” which drives chondrogenic differentiation [43–45]. According to our data, further optimization of differentiation media, aggregate priming, and a longer duration of the study, will be required to achieve a molecular phenotype similar to that of a healthy articular cartilage, as is desired for cartilage tissue engineering.

## Conclusions

In the current study, we successfully designed and fabricated aggregate-encapsulated agarose hydrogels with varied cell concentration and aggregate numbers, resulting in improved GAG content, DNA content, collagen content with increased gene expression of collagen II and aggrecan, and darker IHC staining for collagen and aggrecan compared to the CS control groups. Additionally, significant improvement in performance compared to the cell suspension control groups suggested that the aggregate approach could be a new way of looking at cells used for cartilage regeneration. The aggregate technique presents a simple, robust method of presenting cells as clusters, which, due to inherent interaction and cell-cell communication, and may prove to be a better choice than the conventional cell-suspension-in-hydrogel approach for cell-encapsulated hydrogel approaches for cartilage tissue engineering in the future.

## Supporting Information

S1 File Experimental design. The analyses used for cell viability, biochemical content, gene expression, and immunohistochemistry. (0, 2, and 3 refer to the time points (week) when a given outcome analysis was performed).
(DOCX)
