# Supplementary material for: Stem Cells in Aggregate Form to Enhance Chondrogenesis in Hydrogels
Source: PLoS One. 2015 Dec 31;10(12):e0141479. doi: 10.1371/journal.pone.0141479 (PMC4697858; doi:10.1371/journal.pone.0141479)

**Supplemental Table 1: Experimental design.** The analyses used for cell viability, biochemical content, gene expression, and immunohistochemistry. (0, 2, and 3 refer to the time points (week) when a given outcome analysis was performed).


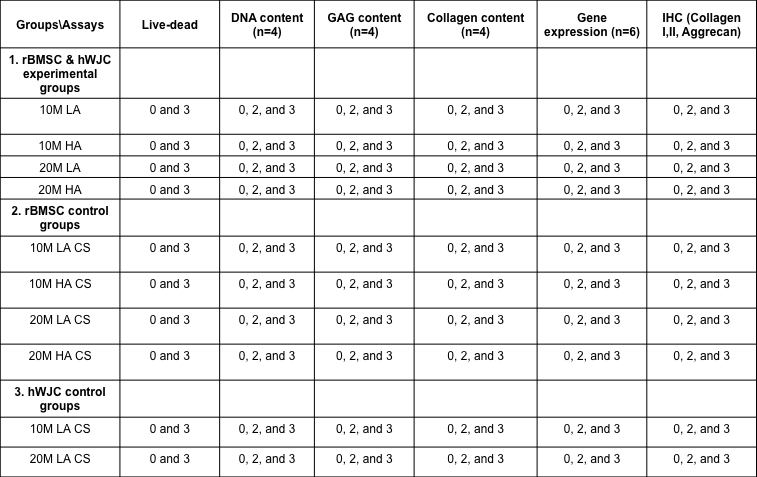

Supplement: S1 File — The analyses used for cell viability, biochemical content, gene expression, and immunohistochemistry. (0, 2, and 3 refer to the time points (week) when a given outcome analysis was performed). (DOCX) [file pone.0141479.s001.docx]
